# Computational Scale-Up for Flood Fed/Starve Fed Single Screw Extrusion of Polymers

**DOI:** 10.3390/polym14020240

**Published:** 2022-01-07

**Authors:** Andrzej Nastaj, Krzysztof Wilczyński

**Affiliations:** Faculty of Mechanical and Industrial Engineering, Polymer Processing Department, Warsaw University of Technology, Narbutta 85, 02-524 Warsaw, Poland; a.nastaj@wip.pw.edu.pl

**Keywords:** polymers, extrusion, scale-up, optimization

## Abstract

A novel scaling-up computer system for single screw extrusion of polymers has been developed. This system makes it possible to scale-up extrusion process with both starve feeding and flood feeding. Each of the scale-up criteria can be an objective function to be minimized, represented by single values or functional dependencies over the screw length. The basis of scaling-up is process simulation made with the use of the GSEM program (Global Screw Extrusion Model). Scaling-up is performed using the GASES program (Genetic Algorithms Screw Extrusion Scaling) based on Genetic Algorithms. Scaling-up the extrusion process has been performed to increase extrusion output according to the scaling-up criteria defined by the single parameters of unit energy consumption, polymer plasticating rate and polymer temperature, as well as by the process parameters profiles of the temperature and plasticating. The global objective function reached the lowest value for the selected process parameters, and extrusion throughput was significantly increased.

## 1. Introduction

Computer modeling is widely used for designing polymer processing. However, CAD/CAE systems, while useful, do not make it possible to optimize the process. Extrusion optimization is a complex issue due to the multiplicity of potential optimization criteria, often contradictory, as well as a very large number of process data: material data, geometry, and operation parameters.

Optimization consists in creating a multidimensional response space of process parameters based on input parameters and searching for extreme values in this space, maximum or minimum. Data for optimization can be obtained on the basis of experimental or simulation tests, but optimization based on simulation data is more effective.

There are many different optimization methods which can be classified into: analytical, stochastic, and enumerative [1,2,3,4,5,6,7,8,9,10]. A characteristic feature of analytical methods is using the gradient of objective function while searching the optimal solution. These methods can be used when derivatives of the objective function are available and when this function is continuous. Furthermore, the application of these methods is limited to unimodal objective functions. Enumerative methods consist in searching the extremes by browsing successively through all possible points of the finite search space. These methods allow to find a global extremum of the objective function. However, their disadvantage is the huge computational cost in case of multidimensional and complex search spaces. Stochastic methods are completely different class of optimization methods. A characteristic feature of these is the use of random mechanisms for searching the extremes of the objective function. Stochastic methods include: simulated annealing and evolutionary computation. Simulated annealing is a probabilistic technique for approximating the global optimum of a given function. It is often used when the search space is discrete as well as in the cases when finding the approximate global optimum is more important than finding the exact local optimum in a fixed time. Evolutionary computations, which are the basic approach of stochastic optimization, consist in searching the extremum of the objective function analogously to the evolution processes in nature and inheritance mechanisms. This is a zeroth-order stochastic method which means that only the values of the objective function are required for searching the extremes. This allows to solve a wide range of optimization tasks where the objective function may be multimodal, discontinuous, non-differential, non-stationary, multivariate, etc. The most widely known evolutionary method are Genetic Algorithms [11,12,13,14,15].

Optimization methods based on Genetic Algorithms are of particular importance in the case of polymer extrusion [16,17,18,19,20,21,22,23,24,25,26].

In general, Genetic Algorithms are characterized by the following features [11]:

- Parameters of the optimization task are processed in the coded form,
- Searching the solution of the optimization task is performed from a randomly selected population, which avoids local extremes,
- The rules of selection of population are probabilistic,
- A new area of searching is determined using previous experiences,
- Only the values of the objective function are required for searching the extremes, the derivatives are not needed.

These methods have been used to optimize most of extrusion processes, i.e., classical flood fed single screw extrusion [16,17,18,19,20], co-rotating extrusion [21,22,23], and starve fed single screw extrusion [24,25,26]. There is a lack of optimization studies on counter-rotating extrusion, although the appropriate mathematical models of this process are available [27,28,29].

An important method of designing physical processes is scaling, i.e., changing the scale of the process according to the selected criteria while maintaining the parameters of the scaled process at a level as close to the parameters of the reference process as possible.

When scaling-up the extrusion process we define the screw geometry and operating conditions of an extruder under designing (the target extruder) that should replicate the operation of the reference extruder. Scaling-up enables to design large extruders based on the studies on the laboratory scale.

Over the years, a number of various scaling-up concepts were proposed. These were discussed in books [30,31,32,33,34,35,36] and in a number of papers.

Most scaling-up concepts were based on the analytical models and consisted in correlating the large and small primary scaling parameters (screw diameter, screw length, screw channel depth, and screw rotational speed) in terms of the exponent of the ratio of the target screw diameter and the reference screw diameter

(1) $$\frac{X_{2}}{X_{1}}={\left(\frac{D_{2}}{D_{1}}\right)}^{s}$$

where *X*_1_, *X*_2_ are the small and large parameter; *D*_1_, *D*_2_ are the small and large screw diameter; *s* is the scaling-up factor.

Carley and McKelvey [37] were the first to scale-up the extrusion process. They considered the metering section of the screw, and proposed to increase the screw channel depth and width in proportion to the ratio of screw diameters, while maintaining the screw rotational speed constant. Later, several other scaling-up concepts were presented [38,39,40,41,42,43,44,45,46,47,48]. Pearson [42], as the first, performed the full extrusion scaling-up analysis considering the solid transport, polymer plasticating and melt flow. The acceptable scale-up was obtained when the numbers of Graetz, Brinkman, and Nahme were constant in the individual zones of the screw. The advantage of this concept was the balanced solid transport, polymer plasticating, and melt flow, the constant specific power consumption, and the power law dependence of the primary parameters.

Rauwendaal [30,46] analyzed and compared the existing scale-up methods and noticed in most cases the unbalanced solids and melt conveying rates and the excessive viscous dissipation, and confirmed a lack of generality of these methods.

This analysis was extended by Covas and Cunha [49] who concluded the available scale-up methods:

- Can tackle the single scale-up criteria only, e.g., plasticating rate or pumping rate and the single step of the process, e.g., polymer plasticating or melt flow;
- Can take into account only a few geometry or process parameters, e.g., screw rotational speed, screw diameter, screw channel depth, screw length;
- Use the simple mathematical models;
- Are not flexible.

Thus, the more effective scaling-up methods are needed which would use the accurate mathematical models, and would allow:

- To consider simultaneously several criteria;
- To select the single parameters or functions as the scale-up criteria;
- To freely choose and define the criteria.

Covas and Cunha concluded [49] that these targets can be achieved by considering the scaling-up as a multi-objective optimization, where the aim is to define the geometry/operating parameters of the target extruder in such a way that the performance parameters of both extruders are as close as possible. The scaling-up aim is to minimize the differences between the selected process parameters of the target and reference extruders. The geometry/operating parameters of the reference extruder are known, and these of the target extruder are looked for.

The implementation of these new scale-up concepts requires:

- Simulating the process to have the response data of the reference extruder at a specific set of input data;
- Defining the scale-up criteria;
- Specifing the fixed parameters of the target extruder;
- Performing the scaling-up by minimizing the differences between the selected parameters of the target and reference extruders.

Selection of the scale-up criteria is fundamental for the quality of scaling-up. Usually, the scale-up criteria include the shear rate, the rates of feeding or pumping, the rate of polymer plasticating, the residence time, and the power consumption as proposed by Rauwendaal [46] and Potente [47].

Covas and Cunha [49] proposed additional criteria:

- Ratio of the total/drag flow rate;
- Specific mechanical energy;
- Pressure variation over the unit screw channel;
- Ratio of the screw length required for melting/total screw length (relative melting length);
- Average shear rate;
- Average shear stress;
- Overall vsicous dissipation;
- Average total strain (WATS), which is the measure of mixing degree.

Most of the scale-up criteria are represented by single values but, in some cases, it might have a sense to consider the profiles of them over the screw length. A good example is the solid bed profile (SBP) which is the ratio of the solid bed width to the screw channel width.

Each of the scale-up criteria may be considered as an objective function *F_i_* to be minimized, for the single values or functional dependencies, in the form [49]

(2) $$F_{i}=\frac{\left|C_{i}-C_{i}^{r}\right|}{C_{i}^{r}}$$

(3) $$F_{i}=\frac{\sum_{k-1}^{K}\frac{\left|C_{i,k}-C_{i,k}^{r}\right|}{C_{i,k}^{r}}}{K}$$

where *F_i_* is the *i*-criterion fitness; $C_{i}$, $C_{i}^{r}$ are the single values of the *i*-criterion for the target extruder and reference extruder; $C_{i,k},$ $C_{i,k}^{r}$ are the values of the *i*-criterion at the *k*-location over the screw length for the target extruder and reference extruder.

The simple way to do a multi-objective optimization is to take into account the global objective function that includes individual objectives with the use, for example, simple scalar function as was done by Covas and Cunha in optimization of extrusion [16,17,18,21,22,23].

Using this approach, Covas and Cunha [49] carried out the scale-up of conventional flood fed single screw extrusion in terms of operating conditions and in terms of screw geometry, as well as they performed the complete scale-up in terms of both operating conditions and screw geometry. This approach allows different criteria to be taken into account at the same time and their relative importance to be considered. The multi-objective scale-up is more efficient than the scale-up based on the single process response because the optimizing procedure finds the solutions that satisfy simultaneously various criteria.

Recently, Vergnes et al. [50] solved the problem of scaling-up the reactive twin screw extrusion, and concluded that the scale-up methods based on the diameter ratios are ineffective as soon as the complex phenomena, like chemical reactions, are involved into the process.

The use of optimization methods for scaling-up, including Multi-Objective Evolutionary Algorithms (MOEA), were also proposed by Covas and Cunha [51,52,53].

Summarizing, scaling-up the extrusion process based on the process modeling is limited, up to now, to the classical single screw extrusion with flood feeding and to the co-rotating extrusion [49,50,51,52,53,54]. The state-of-the-art was recently presented in the review paper [55]. Very recently, the authors [56] presented the first approach to scale-up the single screw extrusion with metered feeding, however, it was limited to the process single responses. Counter-rotating extrusion has not been scaled-up, so far.

In this paper, a novel scaling-up computer system for single screw extrusion with both flood feeding and starve feeding is presented. Each of the scale-up criteria can be an objective function to be minimized, represented by single values (Equation (2)) or functional dependencies over the screw length (Equation (3)). The basis of scaling-up is the process simulation made with the use of the GSEM program (Global Screw Extrusion Model) [57,58,59]. Scaling-up is performed using the GASES program (Genetic Algorithms Screw Extrusion Scaling) based on Genetic Algorithms. Examples of scaling-up are presented for both flood fed extrusion and starve fed extrusion to increase the extrusion throughput according to the scale-up criteria defined by the single parameters of unit energy consumption, plasticating rate, and polymer temperature, as well as by the process parameters profiles of temperature and plasticating.

## 2. Extrusion with Flood Feeding and Metered Feeding

**Figure 1:**
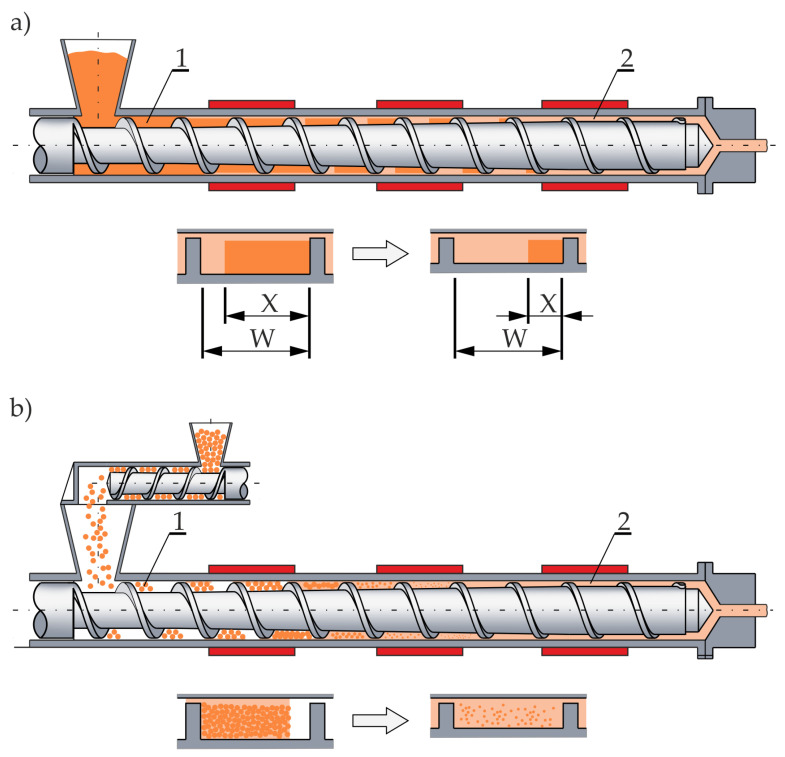
Melting models for single screw extrusion: (**a**) flood fed extrusion, (**b**) starve fed extrusion, (1)—solid conveying, (2)—melt conveying, *X*—solid bed width, *W*—screw channel width.

Extrusion process can be performed with flood feeding or metered feeding. When flood feeding, the screw is fully filled with polymer (Figure 1a), while when metered feeding, the polymer is supplied into the extruder with a dosing device, and the beginning zone of the screw is partially filled with polymer (Figure 1b).

Extrusion with metered feeding, also called extrusion with starving, has some advantages over extrusion with flood feeding [30,60,61,62,63,64,65]. Mixing action is improved, melting is faster, and process control is better. However, the extrusion output is lower.

Single screw extrusion with flood feeding was widely studied, while little studies were performed on extrusion with metered feeding. The state-of-the-art on these issues was discussed in books [30,66,67,68,69], as well as in a number of papers [70,71,72,73,74,75]. Wilczyński et al. reviewed these in [76].

Wilczyński et al. made extensive experimental study [77,78], and proposed the plasticating mechanism and model for single screw extrusion with metered feeding, and built the computer model of the process, SSEM-Starve [57]. Two mechanisms of plasticating were observed, plasticating by heat conduction in the partially filled zone of the screw (Figure 1a), and plasticating by energy dissipation in the fully filled zone of the screw (Figure 1b). Later, the models were proposed for non-conventional screws [58,59], and extrusion of polyblends and polymer composites [79,80,81,82]. All these models were validated experimentally. Rheology and processing of wood polymer composites were discussed in a review paper [83].

Modeling of starve fed extrusion differs substantially from modeling of flood fed extrusion, and different computation algorithms have to be applied, here. This has been discussed in details in [76].

Computation algorithms for single screw extrusion with flood feeding are well known [18,84,85,86]. The computations proceed forward from the hopper to die, and the process operating point is sought, that is the extrusion output and pressure. Computations start for a presumed flow rate, and solid transport, plasticating, and melt flow are calculated. The computed pressure at the die outlet is compared to atmospheric pressure, and the calculations end when these are equal. Otherwise, the flow rate is changed and calculations are iteratively continued until the convergence is achieved.

Computation algorithms for single screw extrusion with metered feeding are less known [57,58,59]. The flow rate is known, and the extrusion pressure is calculated for some presumed polymer temperature. Then, the pressure is calculated backward over the screw. When the pressure diminishes to zero, the starvation starts and the filling of the screw is evaluated. The temperature at the plasticating end is compared to the polymer fusion temperature, and calculations end when these are equal. Otherwise, the polymer temperature is changed and calculations are iteratively continued until the convergence is achieved.

Using these backward algorithms, the models for counter-rotating extruders have been built by the authors [27,28,29], as have been done by other researchers for co-rotating extruders [87,88,89,90]. However, these both models using one-stage melting models are much simpler in execution than the models of starve fed single screw extrusion since the location of melting region is not computed but specified a-priori. In the starve fed single screw extrusion, the location of the transition partially/fully filled screw has to be evaluated in multiple iterative calculations.

**Figure 2:**
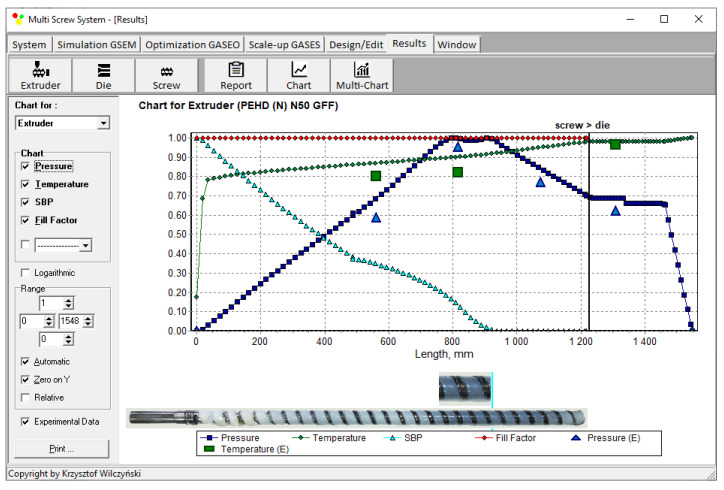
General process characteristics, experiment and simulation with GSEM program—extrusion with flood feeding: *SBP*—solid bed profile, E—measurements.

**Figure 3:**
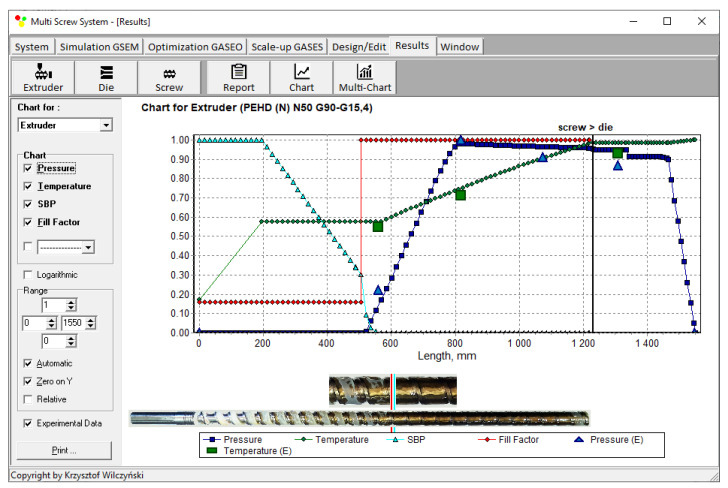
General process characteristics, experiment and simulation with GSEM program—extrusion with metered feeding: *SBP*—solid bed profile, E—measurements.

Recently, the authors built the program GSEM (Global Screw Extrusion Model) [57,58,59,91] for simulating extrusion both with flood feeding and metered feeding. Examples of simulations and experimentations are shown in Figure 2 and Figure 3. The process characteristics include the profiles of pressure and temperature, the solid bed profile, and the screw filling profile. It is clearly seen for extrusion with metered feeding (Figure 3) that the pressure drops to zero when starvation starts. Two stages of melting are also seen. The partially filled zone and fully filled zone are also seen.

## 3. Scaling-Up Procedure

In the study, a scaling-up program GASES (Genetic Algorithms Screw Extrusion Scaling) has been developed. The source of parameters for scaling-up are computations carried out with the program GSEM (Global Screw Extrusion Model) [57,58,59,91].

**Figure 4:**
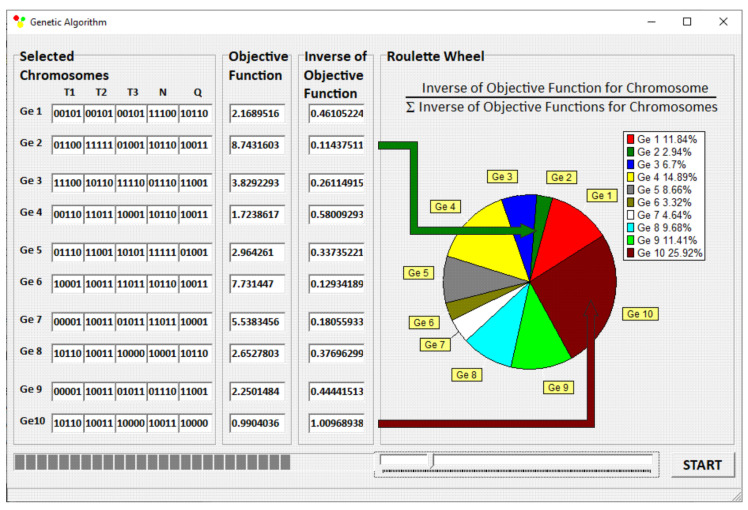
Selection of the initial population and estimation of chromosomes.

GASES scaling-up program, cooperating with the program GSEM, allows for scaling-up the extrusion process with various number of process parameters, with various scale-up criteria specified by the single parameters, e.g., specific energy consumption, the rates of solids conveying, plasticating or pumping, as well as by the process parameters profiles, e.g., plasticating profile, temperature profile, etc. The search accuracy of the response surface is determined by the number of divisions of the data range, which results from the length of writing these numbers in binary form. The length of the binary series is regulated, and its maximum length is 255 characters. This makes it possible to divide the range of each parameter into 2^255^ values. A “roulette wheel” is applied as a method of selection. An operation scheme of the “roulette wheel” is shown in Figure 4. The area of the “roulette wheel” assigned to the individual genotype is inversely proportional to the values of the objective functions generated by the genotypes. The Ge10 genotype has the lowest value of the objective function *F_i_* = 0.9904, i.e., the highest value of the reciprocal of the objective function $\frac{1}{F_{i}}=1.0097$, and covers the surface of the “roulette wheel” equal to 25.92% of the total surface of this. The Ge2 genotype has the highest value of the objective function *F_i_* = 8.9431, i.e., the lowest value of the reciprocal of the objective function $\frac{1}{F_{i}}=0.1144$ covering the surface equal to 2.94% of the total surface of the “roulette wheel”.

**Figure 5:**
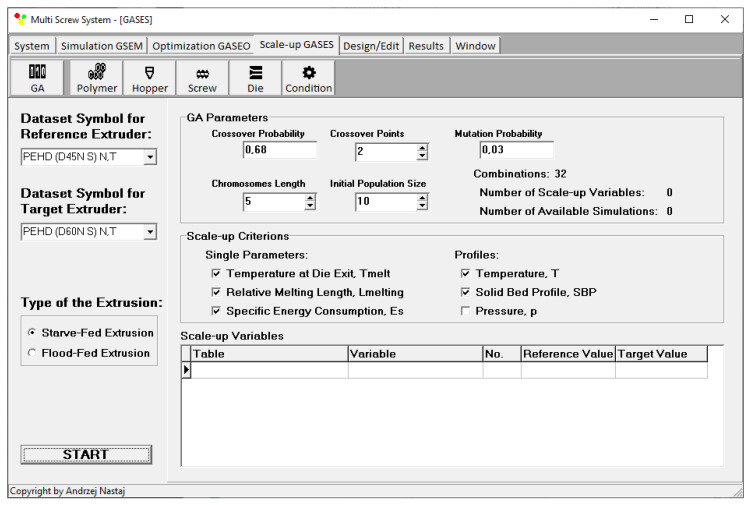
Parameters of the scaling-up procedure.

In the GASES program, scaling-up is defined by the number of scaling variables, the size of initial population, the length of chromosomes, the probability of crossover, the point of crossover, and the probability of mutation. We have not studied the influence of the GA parameters on the results. However, we observed that this influence is rather not important. We observed that GA parameters affect the computation time. We established these parameters based on the literature [11] and our experiences [25,26]. Different weights of the criteria are not available. Scaling-up can be performed for extrusion both with flood feeding and metered feeding. This is depicted in Figure 5.

## 4. Scaling-Up

### 4.1. Research Program

Scaling-up was made for single screw extrusion to increase the process output according to the scale-up criteria defined by the single parameters of unit energy consumption, polymer melting rate, and polymer melt temperature, and by the process parameters profiles of temperature and melting.

The research program included a scale-up of the extrusion process from the level of the reference extruder with the screw of diameter D_r_ = 45 mm to the level of the target extruder with the screw of diameter D_t_ = 60 mm while maintaining the ratio of the length/screw (L_r_/D_r_) = (L_t_/D_t_) = constant. The extrusion with flood feeding and metered feeding were investigated.

A classical three-zone screw of diameter D_r_ = 45 mm, and length/diameter ratio (L_r_/D_r_) = 27 was applied as a reference screw configuration. It has zones of feeding (F), compression (C), and metering (M) with length/diameter ratios equal to (L/D)_F_ = 10.78, (L/D)_C_ = 7.11, and (L/D)_M_ = 9.11. The compression ratio, that is the ratio of the channel depth (H_F_) in the feeding zone to the channel depth in the metering zone (H_M_), CR = H_F_/H_M_, was equal to CR = 2.66 (H_F_ = 8 mm, H_M_ = 3 mm). The die for extrusion of rods of diameter D_die_ = 5 mm was used. Screw geometry of the reference and target extruder are presented in Table 1.

High density polyethylene (HDPE) Rigidex 6070EA (manufactured by BP Chemicals) was used in the study. Material properties are presented in Table 2.

Rheological properties of the polymer (HDPE) were determined with the use of the high-pressure capillary rheometer RG-25 (Göttfert, Buchen, Germany) at temperatures: 180 °C, 190 °C, and 200 °C. The model of Klein was used to describe the viscosity as a function of temperature and shear rate.

(4) $$ln\eta =A_{0}+A_{1}ln\dot{\gamma }+A_{11}ln^{2}\dot{\gamma }+A_{12}Tln^{2}\dot{\gamma }+A_{2}T+A_{22}T^{2}$$

where η is viscosity, $\dot{\gamma }$ is shear rate, *T* is temperature, *A*_0_, *A*_1_, *A*_11_, *A*_12_, *A*_2_, *A*_22_ are parameters of the Klein equation, *A*_0_ = 10.9183, *A*_1_ = −0.2184, *A*_11_ = −0.0368, *A*_12_ = 0.0010, *A*_2_ = −0.0226, *A*_22_ = 0.000021.

### 4.2. Scale-Up of Flood Fed Extrusion 

Scaling-up was performed with reference to the extrusion process, the operation parameters of which were determined as a result of optimization. These parameters were screw rotational speed and barrel temperatures. The optimization of the reference process was carried out according to the criteria of maximum throughput *Q_max_*, minimum unit energy consumption *E_s min_*, and minimum polymer melt temperature at the die outlet *T_melt_*_*min*_, in the range of the screw rotational speed *N* = 20 ÷ 80 rpm and the barrel temperature in the subsequent sections of the extruder: *T*_1_ = 150 °C, *T*_2_ = 150 ÷ 240 °C, *T*_3_ = 150 ÷ 240 °C, *T*_4_ = 150 ÷ 240 °C.

The global objective function was defined as,

(5) $$F_{i\;o}=\sqrt[{3}]{Q_{i\_norm}\cdot E_{s\;i\_norm}\cdot T_{melt\;i\_norm}}$$

The output variables (optimization criteria) were normalized as,

(6) $$Q_{i\_norm}=\frac{Q_{i}-Q_{min}}{Q_{max}-Q_{min}}$$

(7) $$E_{s\;i\_norm}=\frac{E_{j\;i}-E_{j\;min}}{E_{j\;max}-E_{j\;min}}$$

(8) $$T_{melt\;i\_norm}=\frac{T_{melt\;i}-T_{melt\;min}}{T_{melt\;max}-T_{melt\;min}}$$

where *F_i_*_o_ is a global objective function, *Q_i_norm_* is a normalized flow rate, *E_s i_norm_* is a normalized specific energy consumption, *T_melt i_norm_* is a normalized melt temperature at die outlet, *i* is a number of the next value from the data set.

The highest value of an objective function was obtained at the screw rotational speed *N* = 79.53 rpm and the barrel temperatures: *T*_1_ = 150 °C, *T*_2_ = 192.51 °C, *T*_3_ = 180.47 °C, *T*_4_ = 180.47 °C. These optimal parameters, according to the assumed optimization criteria of maximum throughput, minimum unit energy consumption, and minimum polymer melt temperature at the die outlet, correspond to the process output parameters of mass flow rate *Q* = 27.10 kg/h, unit energy consumption: *E_s_* = 489.36 kJ/kg, polymer temperature *T_melt_* = 254.88 °C, and the relative “melting length” *L_melting_* = 0.796, i.e., the ratio of the screw length necessary for melting of polymer to the total screw length.

**Figure 6:**
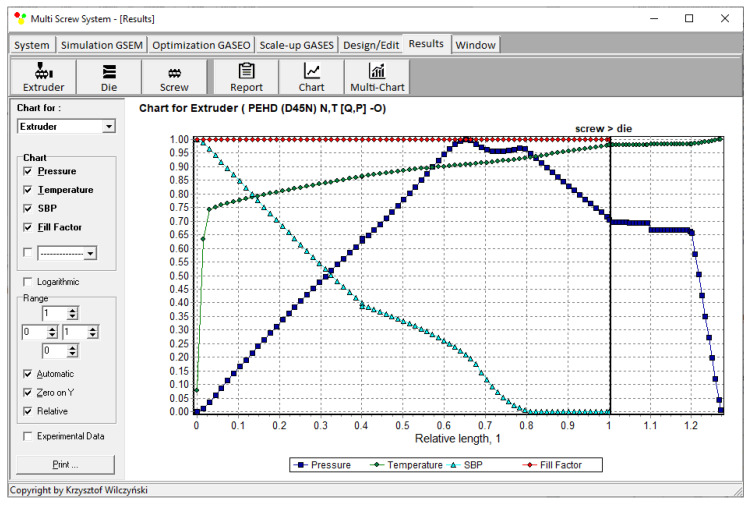
Extrusion with flood feeding: process characteristics (reference extruder) at optimal parameters.

Simulations for the reference extruder at the optimal operation parameters are presented in Figure 6 as a dimensionless process characteristics which includes the profiles of pressure and temperature, the solid bed profile (*SBP*), and the profile of screw filling (FF).

With regard to such an optimized reference process, the extrusion scaling-up was performed in the same range of input data of the screw speed *N* = 20 ÷ 80 rpm, and the barrel temperature in the subsequent sections of the extruder: *T*_1_ = 150 °C, *T*_2_ = 150 ÷ 240 °C, *T*_3_ = 150 ÷ 240 °C, *T*_4_ = 150 ÷ 240 °C.

Scaling-up was carried out according to the single-parameter criteria of the unit energy consumption *E*_s_, the polymer melt temperature *T_melt_*, and the relative “melting length” *L_melting_*, i.e., the ratio of the screw length necessary for polymer melting to the screw length, and to the functional criteria of the temperature profile and the plasticating profile, i.e., the solid bed profile (*SBP*).

The global objective function was defined as,

(9) $$F_{i\;s}=\left|1-\frac{E_{s\;A}}{E_{s\;B_{i}}}\right|+\left|1-\frac{T_{melt_{A}}}{T_{melt\;B_{i}}}\right|+\left|1-\frac{L_{melting\;A}}{L_{melting\;B_{i}}}\right|+\frac{\sum_{k=1}^{n}\left|1-\frac{T_{A\;k}}{T_{B\;ik}}\right|}{n}+\frac{\sum_{k=1}^{n}\left|1-\frac{SBP_{A\;k}}{SBP_{B\;ik}}\right|}{n}$$

where *F_i s_* is the global objective function for scale-up, *E_s A_* is the specific energy consumption for reference extruder, *E_s Bi_* is the specific energy consumption for target extruder, *T_melt_*_*A*_ is the melt temperature at die outlet for reference extruder, *T_melt Bi_* is the melt temperature at die outlet for target extruder, *L_melting A_* is the relative melting length for reference extruder, *L_melting Bi_* is the relative melting length for target extruder, *T_A_* is the melt temperature in reference extruder, *T_B i_* is the melt temperature in target extruder, *SBP_A_* is the polymer melting profile for reference extruder, *SBP_Bi_* is the polymer melting profile for target extruder, *i* is a number of the next value from the data set, *n* is a number of the next value in the profile.

**Figure 7:**
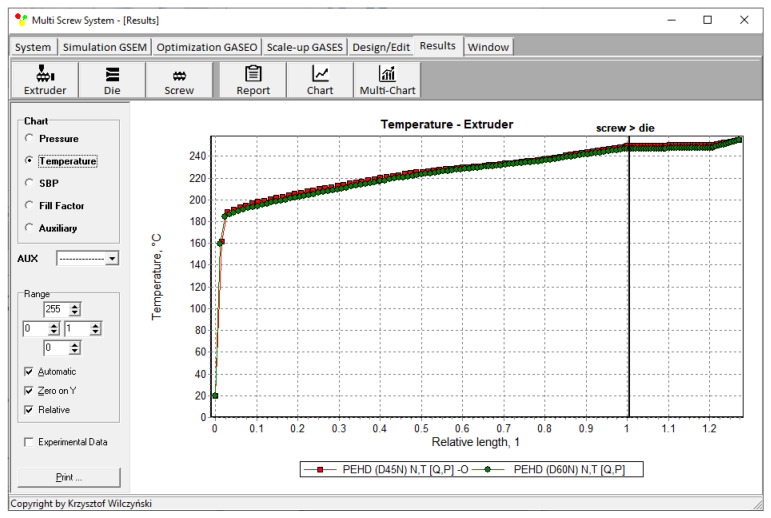
Flood fed extrusion: temperature profile for the reference (red line) and target (green line) extruder.

**Figure 8:**
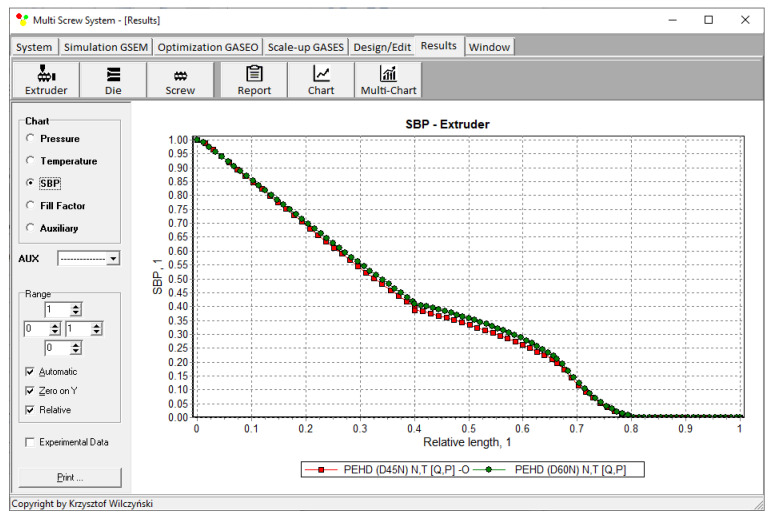
Flood fed extrusion: melting profile (*SBP*) for the reference (red line) and target (green line) extruder.

The results of scaling-up are presented in Table 3, and Figure 7 and Figure 8. The lowest value of the objective function (Equation (9)), i.e., the minimum discrepancy between the variables of the reference and target process, was obtained at the screw speed *N* = 57.14 rpm for the barrel temperatures: *T*_1_ = 150 °C, *T*_2_ = 200 °C, *T*_3_ = 197.85 °C, *T*_4_ = 190.71 °C. These parameters correspond to the process output parameters of the flow rate *Q* = 44.20 kg/h, the unit energy consumption *E*_s_ = 457.76 kJ/kg, the polymer melt temperature *T_melt_ =* 254.93 °C, and the relative “melting length” *L_melting_ =* 0.793. The differences between the parameters of the reference process and the target process are small (Table 3). Thus, it can be concluded that these processes are similar in terms of the selected criteria. The profiles of temperature and melting are also similar which is clearly presented in Table 3 and Figure 7 and Figure 8. By increasing the scale of the process, a significant increase in the extrusion throughput was obtained (63.10%).

### 4.3. Scale-Up of Starve Fed Extrusion 

Scaling-up was performed with reference to the extrusion process, the operation parameters of which were determined as a result of optimization. These parameters were screw rotational speed and barrel temperatures. The optimization of the reference process was carried out according to the criteria of maximum throughput *Q_ST max_*, minimum unit energy consumption *E_s ST min_*, and minimum polymer melt temperature at the die outlet *T_melt ST min_*., in the range of the screw rotational speed *N* = 20 ÷ 80 rpm, the barrel temperature in the subsequent sections of the extruder: *T*_1_ = 150 °C, *T*_2_ = 150 ÷ 240 °C, *T*_3_ = 150 ÷ 240 °C, *T*_4_ = 150 ÷ 240 °C, and the feeding rate *Q*_ST_ = 27.0 ÷ 31.5 kg/h.

The global objective function was defined as,

(10) $$F_{ST\;i\;o}=\sqrt[{3}]{Q_{ST\;i\_norm}\cdot E_{j\;ST\;i\_norm}\cdot T_{melt\;ST\;i\_norm}}$$

The output variables (optimization criteria) were normalized as,

(11) $$Q_{ST\;i\_norm}=\frac{Q_{ST\;i}-Q_{ST\;min}}{Q_{ST\;max}-Q_{ST\;min}}$$

(12) $$E_{s\;ST\;i\_norm}=\frac{E_{s\;ST\;i}-E_{s\;ST\;min}}{E_{s\;ST\;max}-E_{s\;ST\;min}}$$

(13) $$T_{melt\;ST\;i\_norm}=\frac{T_{melt\;ST\;i}-T_{melt\;ST\;min}}{T_{melt\;ST\;max}-T_{melt\;ST\;min}}$$

where *F_i o_* is a global objective function, *Q_ST i_norm_* is a normalized flow rate, *E_s ST i_norm_* is a normalized specific energy consumption, *T_melt ST i_norm_* is a normalized melt temperature at die outlet, *i* is a number of the next value from the data set.

The highest value of the objective function was obtained at the screw rotational speed *N* = 80 rpm and the barrel temperatures: *T*_1_ = 150 °C, *T*_2_ = 234.33 °C, *T*_3_ = 228.66 °C, *T*_4_ = 222.99 °C, and the feeding rate *Q_ST_* = 27.32 kg/h. These optimal parameters, according to the assumed optimization criteria of maximum throughput, minimum unit energy consumption, and minimum polymer melt temperature at the die outlet, correspond to the process output parameters of mass flow rate *Q_ST_* = 27.32 kg/h, unit energy consumption *E_s_*_*ST*_ = 453.84 kJ/kg, polymer temperature *T_melt ST_* = 223.31 °C, and the relative “melting length” *L_melting_* = 0.617, i.e., the ratio of the screw length necessary for melting of polymer to the total screw length.

**Figure 9:**
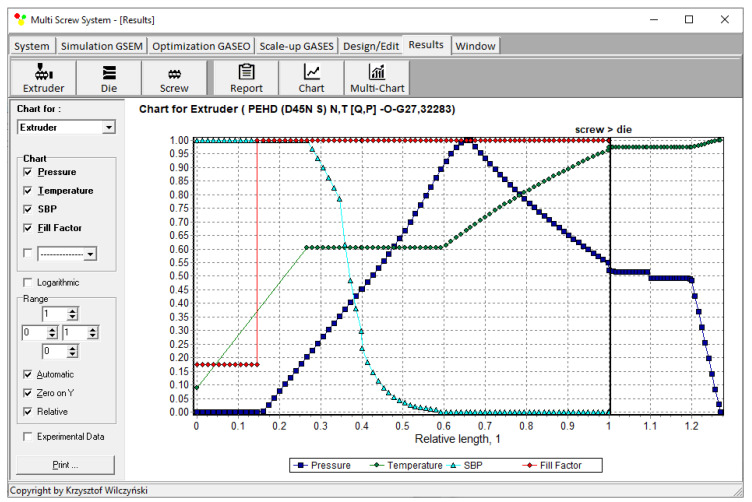
Extrusion with metered feeding: process characteristics (reference extruder) at optimal parameters.

Simulations for the reference extruder at the optimal operation parameters are presented in Figure 9 as a dimensionless process characteristics which includes the profiles of pressure and temperature, the solid bed profile (SBP), and the profile of screw filling (FF).

With regard to such an optimized reference process, the extrusion scaling-up was performed in the same range of input data of the screw speed *N* = 20 ÷ 80 rpm, the barrel temperature in the subsequent sections of the extruder: *T*_1_ = 150 °C, *T*_2_ = 150 ÷ 240 °C, *T*_3_ = 150 ÷ 240 °C, *T*_4_ = 150 ÷ 240 °C, and the feeding rate *Q*_ST_ = 36 ÷ 42 kg/h.

Scaling-up was carried out according to the single-parameter criteria of the unit energy consumption *E_s ST_*, the polymer melt temperature *T_melt ST_*, and the relative “melting length” *L_melting ST_*, i.e., the ratio of the screw length necessary for polymer melting to the screw length, and to the functional criteria of the temperature profile and the plasticating profile, i.e., the solid bed profile (*SBP*).

The global objective function was defined as,

(14) $$F_{ST\;i\;s}=\left|1-\frac{E_{S\;ST\;A}}{E_{S\;ST\;B_{i}}}\right|+\left|1-\frac{T_{\mathrm{melt}\;ST\;A}}{T_{\mathrm{melt}\;ST\;B_{i}}}\right|+\left|1-\frac{L_{\mathrm{melting}\;ST\;A}}{L_{\mathrm{melting}\;STB_{i}}}\right|+\frac{\sum_{k=1}^{n}\left|1-\frac{T_{ST\;A\;k}}{T_{ST\;B\;ik}}\right|}{n}+\frac{\sum_{k=1}^{n}\left|1-\frac{SBP_{ST\;A\;k}}{SBP_{ST\;B\;ik}}\right|}{n}x$$

where *F_ST i s_* is the global objective function for scale-up, *E*_s ST A_ is the specific energy consumption for reference extruder, *E_s ST Bi_* is the specific energy consumption for target extruder, *T_melt ST A_* is the melt temperature at die outlet for reference extruder, *T_melt ST Bi_* is the melt temperature at die outlet for target extruder, *L_melting ST A_* is the relative melting length for reference extruder, *L_melting ST Bi_* is the relative melting length for target extruder, *T_ST A_* is the melt temperature in reference extruder, *T_ST Bi_* is the melt temperature in target extruder, *SBP_ST A_* is the polymer melting profile for reference extruder, *SBP_ST Bi_* is the polymer melting profile for target extruder, *i* is a number of the next value from the data set, *n* is a number of the next value in the profile.

**Figure 10:**
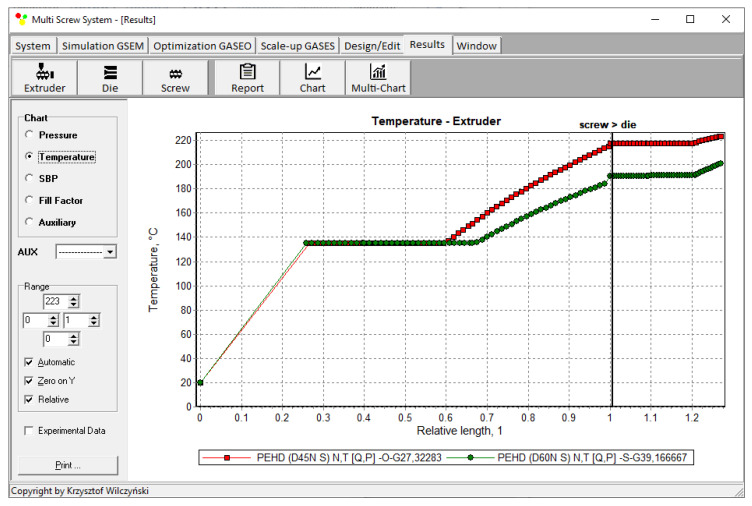
Starve fed extrusion: temperature profile for the reference (red line) and target (green line) extruder.

**Figure 11:**
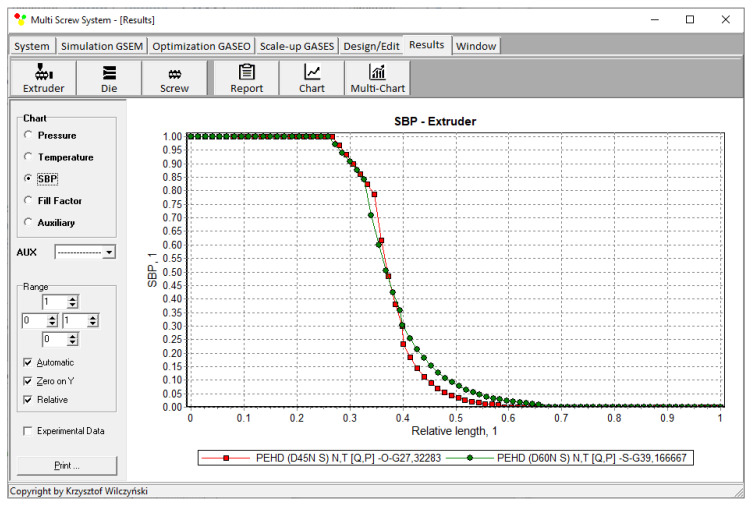
Starve fed extrusion: melting profile (*SBP*) for the reference (red line) and target (green line) extruder.

The results of scaling-up are presented in Table 4, and Figure 10 and Figure 11. The lowest value of the objective function (Equation (9)), i.e., the minimum discrepancy between the parameters of the reference and target process, was obtained at the screw speed *N* = 47.67 rpm for the barrel temperatures: *T*_1_ = 150 °C, *T*_2_ = 204 °C, *T*_3_ = 194.33 °C, *T*_4_ = 193 °C, at the feeding rate *Q_ST_* = 39.17 kg/h. These parameters correspond to the process output parameters of the unit energy consumption *E_s ST_* = 360.32 kJ/kg, the polymer melt temperature *T_melt ST_ =* 200.67 °C, and the relative “melting length” *L_melting ST_ =* 0.571. The differences between the parameters of the reference process and the target process are small (Table 4). Thus, it can be concluded that these processes are similar in terms of the selected criteria. The profiles of temperature and melting are also similar which is clearly presented in Table 4 and Figure 10 and Figure 11. By increasing the scale of the process, a significant increase in the extrusion throughput was obtained (43.37%).

## 5. Conclusions

A novel scaling-up computer system for single screw extrusion of polymers has been developed. This system makes it possible to scale-up extrusion process with both starve feeding and flood feeding. Each of the scale-up criteria can be an objective function to be minimized, represented by single values or functional dependencies over the screw length. Scaling-up the extrusion process has been performed to increase extrusion output according to the scaling-up criteria defined by the single parameters of energy unit consumption, polymer melting rate, and polymer temperature, as well as by the process parameters profiles of temperature and melting. The global objective function reached the lowest value for the selected process parameters, and extrusion throughput was significantly increased. The use of functional scaling-up criteria in addition to the single-parameter criteria increased the accuracy of scaling-up.

It is worth noticing that the surface of the “roulette wheel” assigned to the individual genotypes is inversely proportional to the objective function values generated by these. When the genotype has the lowest value of the objective function, i.e., the highest value of the reciprocal of the objective function, the largest area of the “roulette wheel” is covered. When the genotype has the highest value of the objective function, i.e., the lowest value of the reciprocal of the objective function, the smallest area of the “roulette wheel” is covered.

So far, there is a lack of optimizing and scaling-up studies on the counter-rotating twin screw extrusion. However, the global models of this process are available. Thus, it seems to be reasonable to apply the Genetic Algorithms to solve this task, as for the co-rotating twin screw extrusion. Defining the screw geometry for the counter-rotating extrusion is similar to that for the co-rotating one, and differs from that for the single screw extrusion. In the former, the screw configuration is determined by choosing the screw elements from a set of elements available and locating them along the screw. In the latter, the geometry variables can vary continuously within a prescribed range.

## Figures and Tables

**Table 1:** Screw geometry.

Screw Geometry		
	Reference	Target
Length of feeding/compression/metering zone	10.78; 7.11; 9.11 turns	10.78; 7.11; 9.11 turns
Diameter of barrel, D_b_	45 mm	60 mm
Screw pitch	45 mm	60 mm
Depth of screw channel in feeding zone, H_F_	8 mm	12 mm
Depth of screw channel in metering zone, H_M_	3 mm	4.5 mm
Flight width	5 mm	5 mm

**Table 2:** Material properties—HDPE Rigidex 6070EA.

Material Properties	
Density—bulk	595 kg/m^3^
Density—solid	951 kg/m^3^
Density—melt	721 kg/m^3^
Polymer-barrel friction factor	0.40
Polymer-screw friction factor	0.25
Heat of fusion	245,000 J/kg
Solid specific heat	2250 J kg^−1^ deg^−1^
Melt specific heat	3000 J kg^−1^ deg^−1^
Thermal conductivity	0.27 W m^−1^ deg^−1^
Melt flow rate (190 °C, 2.16 kg)	7.6 g/10 min

**Table 3:** Results of scaling-up the flood fed extrusion process.

Results of Scaling-Up the Extrusion Process			
	Extruder		
Single Parameter	Reference	Target	Deviation
Specific energy consumption	489.36 kJ/kg	457.76 kJ/kg	6.46%
Relative melting length	0.769	0.793	3.12%
Polymer melt temperature	254.88 °C	254.93 °C	0.02%
Extrusion throughput	27.10 kg/h	44.20 kg/h	63.10%
**Profile**			
Temperature profile			
1.	20.00 °C	20.00 °C	0.00%
2.	128.55 °C	159.16 °C	21.48%
3.	176.15 °C	184.41 °C	4.69%
…	…	…	…
141.	254.48 °C	254.21 °C	0.11%
142.	254.84 °C	254.82 °C	0.00%
*SBP* profile			
1.	1.00	1.00	0.00%
2.	0.99	0.99	0.00%
3.	0.96	0.97	1.04%
…	…	…	…
75.	0.01	0.01	0.00%
76.	0.00	0.00	-

**Table 4:** Results of scaling-up the starve fed extrusion process.

Results of Scaling-Up the Extrusion Process			
	Extruder		
Single Parameters	Reference	Target	Deviation
Specific energy consumption	453.84 kJ/kg	360.32 kJ/kg	20.61%
Relative melting length	0.617	0.571	7.46%
Polymer melt temperature	223.31 °C	200.67 °C	0.02%
Extrusion throughput/Feeding flow rate	27.32 kg/h	39.17 kg/h	43.37%
**Profiles**			
Temperature profile			
1.	20.00 °C	20.00 °C	0.00%
…	…	…	…
21.	132.12 °C	135.00 °C	2.18%
22.	135.00 °C	135.00 °C	0.00%
…	…	…	…
126.	223.26 °C	200.67 °C	10.12%
*SBP* profile			
1.	1.00	1.00	0.00%
…	…	…	…
21.	0.98	0.97	1.02%
22.	0.95	0.94	1.05%
…	…	…	…
46.	0.00	0.00	-

## Data Availability

The data presented in this study are available on request from the corresponding author.
